# Comparison of the Effect of Hydrostatic and Dynamic High Pressure Processing on the Enzymatic Activity and Physicochemical Quality Attributes of ‘Ataulfo’ Mango Nectar

**DOI:** 10.3390/molecules27041190

**Published:** 2022-02-10

**Authors:** Manuel Alejandro Uranga-Soto, Manuel Alejandro Vargas-Ortiz, Josefina León-Félix, José Basilio Heredia, María Dolores Muy-Rangel, Dominique Chevalier-Lucia, Laetitia Picart-Palmade

**Affiliations:** 1 Centro de Investigación en Alimentación y Desarrollo A.C. Coordinación Regional, Culiacan 80110, Mexico; manuel.urangadc18@estudiantes.ciad.mx (M.A.U.-S.); ljosefina@ciad.mx (J.L.-F.); jbheredia@ciad.mx (J.B.H.); 2 CONACYT-CIAD (Centro de Investigación en Alimentación y Desarrollo), Laboratorio de Calidad, Autenticidad y Trazabilidad de los Alimentos, Hermosillo 83304, Mexico; 3 IATE, INRA, Institut AGRO, University Montpellier, 34090 Montpellier, France; dominique.chevalier-lucia@umontpellier.fr (D.C.-L.); laetitia.palmade@umontpellier.fr (L.P.-P.)

**Keywords:** hydrostatic high pressure, high-pressure homogenization, enzymatic activity, color, rheology, mango nectar

## Abstract

The effects of hydrostatic (HHP) and dynamic (HPH) high-pressure treatments on the activity of pectin methylesterase (PME) and polyphenol oxidase (PPO) as well as the physicochemical quality attributes of ‘Ataulfo’ mango nectar were assessed. HHP reduced PME relative activity by 28% at 100 MPa for 5 min but increased PPO activity almost five-fold. Contrarily, HPH did not affect PME activity, but PPO was effectively reduced to 10% of residual activity at 300 MPa and at three passes. Color parameters (CIEL*a*b*), °hue, and chroma were differently affected by each type of high-pressure processing technology. The viscosity and fluid behavior were not affected by HHP, however, HPH changed the apparent viscosity at low dynamic pressure levels (100 MPa with one and three passes). The viscosity decreased at high shear rates in nectar samples, showing a shear-thinning effect. The results highlight how different effects can be achieved with each high-pressure technology; thus, selecting the most appropriate system for processing and preserving liquid foods like fruit beverages is recommended.

## 1. Introduction

In the production of high-quality food products, like fruit-based beverages, enzyme inactivation is considered a requirement for sufficient shelf life. Fruit-based beverages contain indigenous enzymes like pectinases and oxidoreductases, which are considered as the main causes of undesirable changes in color, texture, and other sensory attributes during storage [1,2]. Thermal processing has been so far the most effective preservation technology in the industry for the inactivation of enzymes like pectin methylesterase (EC 3.1.1.11) and polyphenol oxidase (EC 1.10.3.1) in fruit-based beverages, although it affects the nutritional content and sensory attributes of food products [3]. Alternatively, non-thermal processing technologies like hydrostatic high pressure (HHP) or high-pressure homogenization (HPH) are becoming more popular for their advantages over conventional preservation technologies [4,5]

HHP is a technology that does not cause significant changes in molecules because it only affects non-covalent bonds, unlike thermal processing, and it has a superior capacity for the retention of nutrients, nutraceutical compounds, and sensory attributes of foods [6]. The main enzyme inactivation mechanism associated with HHP is the loss of their native tridimensional configuration (tertiary and quaternary structures), which are stabilized by non-covalent bonds. These conformations are disrupted when hydrostatic pressure is applied in an aqueous medium [7]. Unlike HHP, HPH is mostly used for the processing of liquid foods with a very limited range of suspended particle sizes. At a molecular and structural level, the shear stress induced by HPH and the thermal effect by adiabatic heating (temperature increases around 20 °C per 100 MPa) cause disruption of the particles in the food system; hence, molecules like proteins are unfolded and aggregated by the effect of homogenization, which could lead to losing their catalytic activity and increasing the product’s shelf life [8,9,10].

There are numerous reports of the effects of HHP on the enzymatic activity in fruit-based food products, often with variable results in terms of enzyme inactivation. However, HHP in combination with thermal treatments has been evaluated to increase process efficiency for enzyme inactivation in fruit-based food products [11,12,13]. Reports on HPH, by contrast, have been focused on the inactivation of PME in citrus juices [14,15,16]. In terms of a comparison between these technologies, there are scarce reports providing information on HHP and HPH on the same matrix in a similar high-pressure range. HHP has been compared with sonication, thermal treatments, and pulsed electric fields in terms of enzyme inactivation efficiency [17,18,19]. Meanwhile, the direct comparison of these pressure-based processing technologies has been limited to a few studies, such as the one on egg white lysozyme by Tribst et al. [20]. This provides an opportunity for comparing the effect of these technologies on fruit-based products and their attributes.

Considering the importance of the residual activity of enzymes such as PME and PPO in the overall quality and shelf life of fruit-based products, the objective of this study was to compare the effect of HHP and HPH on the activity of both enzymes using ‘Ataulfo’ mango nectar as a study model. The physicochemical parameters often associated with the activity of these enzymes, such as color and viscosity, were also evaluated, as they are important quality attributes in this type of product.

## 2. Results and Discussion

### 2.1. Effect of High Hydrostatic and Dynamic Pressure on the Relative Enzymatic Activity of Pectin Methylesterase

Concerning the effect of high hydrostatic pressure processing on PME relative activity, the Analysis of Variance (ANOVA) showed that all the experimental factors, with the exception of hydrostatic pressure level, were significant (*p* ≤ 0.05), with a significant interaction between the hydrostatic pressure level and the processing time experimental factors. Our results showed an interesting trend, where the relative enzymatic activity of PME was reduced to 72.6% at 100 MPa and 5 min, in contrast with higher pressure levels and processing times, where activity remained above 90% (Table 1). For analyzing the effect of dynamic pressure processing on the relative enzymatic activity of PME, the outlet temperature after the high-pressure valve of the homogenization system (Table 2) was included as a covariate in the ANOVA. Adjusting the terms of the factorial regression model showed that the dynamic pressure level and outlet temperature were significant as a linear model (*p* ≤ 0.05). The Variance Inflation Factor (VIF = 15.31) showed a high correlation between dynamic pressure level and outlet temperature on PME relative activity, despite the fact that it was mostly unaffected and partially increased (up to 119.9%) by dynamic pressure processing. In comparison, neither of the high-pressure processing systems was efficient for reducing PME activity under the studied processing conditions. Although the outlet temperature was significantly correlated to dynamic pressure, the holding times at those temperatures during processing might not be sufficient to synergize with the pressure to reduce PME activity. It is commonly stated in the literature that PME is a highly baroresistant enzyme, as it can withstand hydrostatic pressure levels higher than 600 MPa [21]. Previously reported results of the HHP effect of PME in mango nectar by Bermudez-Aguirre et al. [22] showed that at 345 MPa, activity was reduced by 45%, but at 414 MPa, PME increased its activity. Regarding the effect of high dynamic pressure on PME, Welti-Chanes et al. [23] reported 75% inactivation of PME in orange juice treated at 250 MPa with an initial temperature of 45 °C. Their results might suggest that the thermal effects from adiabatic heating were mainly responsible for the higher level of inactivation, as opposed to our results, where we kept the initial temperature at 10 °C to reduce thermal effects as much as possible. Similarly, Velázquez-Estrada et al. [16], in orange juice, achieved 95% inactivation of PME at 300 MPa, with a final temperature of 95 °C. Likewise, Navarro et al. [15] reported 10% residual PME activity in clementine juice processed at 150 MPa and 68 °C. These previous works support our claims that thermal effects might be more pronounced and impactful than dynamic pressurization on enzyme inactivation.

### 2.2. Effect of High Hydrostatic and Dynamic Pressure on Relative Enzymatic Activity of Polyphenol Oxidase

Regarding the effect of high hydrostatic pressure processing on PPO relative activity, none of the experimental factors were significant (*p* > 0.05). As shown in Table 1, the relative enzymatic activity of PPO was highly increased between 337.0 and 481.3% among all treatments. The ANOVA of the effect of dynamic pressure processing on the relative activity of PPO showed that outlet temperature was significantly correlated (*p* ≤ 0.05, VIF = 72.78) with dynamic pressure level and possibly had a greater effect on PPO activity, as pressure level and the number of passes were not significant (*p* = 0.067 and 0.055, respectively). As observed in Table 1, the relative enzymatic activity of PPO increased at 100 and 200 MPa; however, processing at 300 MPa for one and three passes reduced the relative activity to 26.7 and 10.7%, respectively. To discard any unwanted effect in enzyme activity due to possible variations in protein content between samples, the average protein content in the samples treated by HHP and HPH, respectively, was 10.7 ± 1.0 and 9.9 ± 0.4 mg/mL, as quantified by the BCA assay. There was no significant difference between the protein content of the nectar samples; hence, the differences in enzymatic activity between processing conditions might not relate to changes in protein content. In comparison with HHP, the reduction of PPO activity in HPH treated samples could be attributed to the possible synergy between high dynamic pressure and the thermal effects due to adiabatic heating in the system, since temperatures of 65 and 73 °C were reached at the HP valve outlet after one and three passes, respectively, and their correlation was statistically significant. However, due to the function of the cooling system in the HPH device, the treated samples stayed at these high temperatures for holding times of less than one second. Our results suggest that HPH might be more effective than HHP for reducing the activity of PPO in fruit-based beverages, as long as the dynamic pressure synergizes with high temperatures. In the literature, PPO is considered resistant to HHP at 100–600 MPa, and some cases of increased activity have been observed within these pressure levels [24]. Unlike PME, there are fewer reports of PPO inactivation by high dynamic pressure. Bot et al. [25] reported a reduction of PPO activity in apple juice by 50% after 10 passes at 150 MPa. These conditions, however, are not feasible in an operational environment for pilot or commercial-scale production. In addition, Marszalek et al. [26] reported that the combination of dynamic pressure and heat increases enzyme inactivation at >200 MPa in dynamic pressure systems, which supports our results showing how PPO activity was reduced significantly at 300 MPa for one and three passes due to the correlated effect of dynamic pressure and high outlet temperature from adiabatic heating.

### 2.3. Effect of High Hydrostatic and Dynamic Pressure on Color Parameters

**Figure 1:**
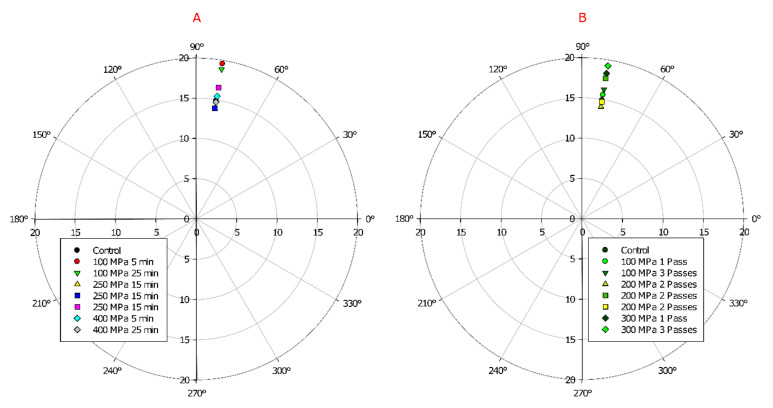
Polar plot of °hue vs. chroma of ‘Ataulfo’ mango nectar processed by high pressure: (**A**) hydrostatic pressure, (**B**) dynamic pressure.

In relation to the effect of high hydrostatic pressure processing on the color parameters of mango nectar, the ANOVA showed that the experimental factors were not significant, except for the two-way interaction between hydrostatic pressure level and processing time, which significantly affected a* values (*p* ≤ 0.05). As shown in Table 3, the samples processed by HHP retained the luminosity (L*) and b* values, while a* increased slightly in comparison to the control sample. The total color difference values (ΔE) concerning the JND (Just Noticeable Difference) ranges indicated that only an experienced observer could notice differences between samples [27]. The effect of processing conditions on °hue and chroma is shown in Figure 1A. The °hue of samples is near to the yellow color (90°), and the highest chroma value was observed in samples treated at 100 MPa for 5 min. The lower chroma values indicate a duller color saturation when combined with the color tone (°hue), and considering the slight decrease in L* in HHP samples, these effects might be related to the highly increased activity of PPO after processing. It is known in the literature that HHP is a preservation technology that efficiently retains sensory attributes like color. Aaby et al. [28] reported good color retention in strawberry puree processed by HHP. Moreover, our results showed that the samples processed by HPH experienced increases in L* and b* values, while a* was slightly reduced compared to the control sample, despite the fact that the ANOVA showed no significant effects from the experimental factors. The effect of HPH on sample color was slightly different from that of HHP, as the ΔE values were higher, indicating a clear difference of color in comparison to the control sample. In addition, the results of °hue and chroma values (Figure 1B) indicated that the mango nectar processed by HPH became lighter in color (higher L* values from Table 3), and the yellow coloration increased as the dynamic pressure level increased. This could be attributed to the reduced PPO activity in HPH processed samples compared to HHP, and the disruptive effect of HPH on cells, as it can lead to the release of pigments into the serum phase of the product [29]. Guan et al. [30] reported an increase in carotenoids from mango juice treated by HPH. These pigments relate to the characteristic yellow coloration of ‘Ataulfo’ mangoes. Kruszewski et al. [31] reported a similar behavior in the color parameters of blackcurrant juice processed by HPH. Processing slightly increased L* and b* values, while a decrease in a* was observed. These effects were attributed to a partial loss of the main pigment (decreased a* values) and non-enzymatic browning (increased b* value). In our results, the increase in b* value is a desirable trait, because carotenoids are the main pigments in ‘Ataulfo’ mangoes and can be associated with a more intense yellow coloration.

### 2.4. Effect of High Hydrostatic and Dynamic Pressure on Rheological Parameters

**Figure 2:**
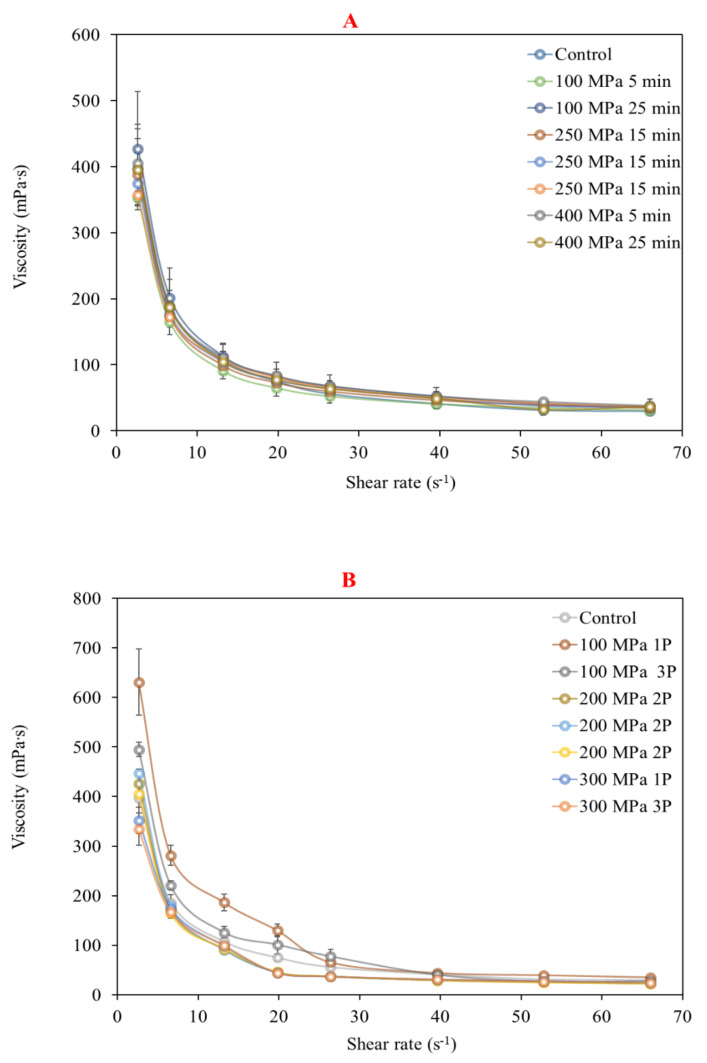
Apparent viscosity as a function of shear rate in mango nectar processed by high pressures: (**A**) hydrostatic pressure, (**B**) dynamic pressure. Bars show standard errors.

The rheological behavior of mango nectar samples processed by HHP and HPH showed a difference between both processing technologies only at the 100 MPa pressure level, especially at low shear rates. The changes in apparent viscosity as a function of the shear rate of the samples processed by HHP or HPH are presented in Figure 2. All nectar samples presented a shear-thinning behavior, associated with pseudo-plastic non-Newtonian fluids. The rheology of nectar samples after HHP compared to the control sample was not significantly affected, independently of the processing conditions (hydrostatic pressure level and processing time). Liu et al. [32] reported that HHP had no significant effect on the viscosity of mango nectars, unlike a high-temperature short-time treatment (HTST), which decreased viscosity. The effects in sample rheology in HHP may depend on the relation between the pressure level and holding time with the total soluble solids content of the processed sample [33]. In our results, HPH had a greater impact on fluid behavior and viscosity than HHP. An interesting phenomenon was observed in nectar samples treated by HPH at 100 MPa (one and three passes) and low shear rates, as their viscosity was higher than that of the control sample. In contrast, samples treated at 300 MPa by HPH at the same shear rates did not show the same effect, as apparent viscosity was significantly lower than the control samples at high shear rates (Table 4). This behavior could be attributed to the effect of HPH on pectins and the other polysaccharides present in mango pulp, as it might reduce the apparent viscosity of the serum phase of the nectar, altering consistency and texture [7]. In addition, HPH modifies the particle size and distribution, which could increase the particle–particle and particle–serum interactions, resulting in variable pulp stability [34]. It is important to mention that the effects on viscosity could also be product-dependent, as Leite et al. [35] reported that HPH reduced the consistency of apple juice and claimed that the response of each product to HPH could be difficult to predict.

## 3. Materials and Methods

### 3.1. Plant Material

‘Ataulfo’ variety mango fruits were harvested from an orchard in Chiapas, Mexico (latitude 15.285056, longitude −92.698389) at ripeness stage 4 (14–16 °Brix and pH = 4.5–4.7 in pulp). Fruits were washed in a 200 ppm sodium hypochlorite solution. Pulp was separated from the pericarp, packed, sealed in vacuum bags, and stored at −20 °C. Frozen pulp was transferred to the facilities of the UMR IATE of the University of Montpellier to perform the experiments. Mango pulp was stored at −20 °C throughout the experimental phase.

### 3.2. Mango Nectar Formulation

The pulp was thawed overnight at 4 °C and blended in a thermomixer for 120 s at high speed until a smooth paste was formed. The nectar formulation contained 40% *w*/*w* pulp paste diluted with reverse-osmosis-treated water and mixed by magnetic agitation until fully homogenized. No sugar or additives were added. The nectar was filtered through a sieve of 250 µm mesh before high-pressure processing, and the pH, soluble solids (°Brix), and color (CIEL*a*b*) of the untreated filtered nectar (control) were measured.

### 3.3. High Hydrostatic Pressure Processing

Pressurization experiments were carried out in a 1 L capacity high-pressure stainless steel vessel from ACB (Nantes, France), with a maximum operating pressure of 450 MPa. Samples were treated at pressure levels of 100, 250, or 400 MPa for a gradual screening of enzyme activity, with 5, 15, and 25 min processing times and an initial temperature (Ti) = 10 °C, using distilled water as a pressure transmitting fluid. For each batch, 300 mL of nectar was packed in polyvinylidene chloride (PVDC) tubings (26 mm diameter, 50 µm thick, Krehalon, Eygalières, France), sealed with two knots at both ends, and kept at 4 °C for a maximum of 4 h before being processed (control assay). After processing, one set of samples was used for the determination of the enzymatic activity.

### 3.4. High Dynamic Pressure Processing

High-pressure homogenization experiments were carried out in a Stansted Fluid Power UHP homogenization system (Essex, UK), which operates at a maximum dynamic pressure of 350 MPa. Nectar samples were stored in a sampling stainless steel tank at 10 °C and treated at 100, 200, and 300 MPa for 1, 2, and 3 passes. Temperatures were recorded before and after the HP valve. After processing, one set of samples was used for the determination of the enzymatic activity immediately after processing.

### 3.5. Enzymatic Activity Assays

#### 3.5.1. Enzyme Extraction

Nectar samples processed by HHP and HPH were diluted with extraction buffer (0.2 M sodium phosphate pH 6.4, 4% *w*/*w* polyvinylpolypyrrolidone, 1 M NaCl) at a 4:1 buffer-to-sample ratio and mixed at 4 °C for 1 h. Afterward, samples were centrifuged at 37,750× *g* at 4 °C for 30 min in a Sorvall RC 5B Plus (Du Pont, Wilmington, DE, USA) centrifuge [36,37]. The supernatant was collected and used as an extract for enzymatic activity assays. Protein content in enzyme extracts was determined by the bicinchoninic acid (BCA) assay.

#### 3.5.2. Pectin Methylesterase Activity Assay

The activity of pectin methylesterase was measured according to Rodrigo et al. [38], and 0.25 mL of extract was added to 30 mL of substrate solution (0.35% apple pectin, 0.12 mM NaCl pH 7.0) to start the reaction. Then, 0.05 N NaOH was automatically added by a 902 Titrando automatic titrator fitted with an 800 Dosino Unit (Metrohm, Herisau, Switzerland) for a reaction time of 6 min. The activity of PME was calculated according to the following equation:

(1) $$\mathrm{PME}\;\left(U\right)=\frac{V\cdot N\cdot 1000}{V_{s}\cdot t}$$

where

***V*** = volume of NaOH (mL);

***N***= normality of NaOH;

***V_s_*** = sample volume (mL);

***t*** = reaction time (min).

All assays were performed at 25 °C in triplicate.

#### 3.5.3. Polyphenol Oxidase Activity Assay

The activity of polyphenol oxidase was measured according to the method by Palma-Orozco et al. [39]; 50 µL of enzyme extract was added to 1 mL of substrate solution (0.2 M sodium phosphate buffer pH 6.6, 50 mM pyrocatechol) to initiate the reaction in a 1 cm quartz cell. Then, absorbance was measured every 5 s for 5 min in a Unicam UV2 UV-Vis spectrophotometer (ATi Unicam, Cambridge, UK) at 420 nm. PPO activity was calculated from the slope of the initial linear portion of the absorbance vs. time curve and expressed in units per mL of extract. All assays were performed at 25 °C in triplicate.

#### 3.5.4. Relative Enzymatic Activity Calculation

The activities of PME and PPO were calculated as units per mL of extract. For better comparison against the control sample, the values were converted to relative activity regarding the untreated nectar, which was calculated according to the following equation:

(2) $$\mathrm{Relative}\;\mathrm{Enzymatic}\;\mathrm{Activity}\;\left(\%\right)=\frac{A_{s}}{A_{0}}\times 100$$

where

*A_s_* = enzymatic activity in U/mL of the processed sample;

*A_0_* = enzymatic activity in U/mL of the untreated sample.

### 3.6. Color Parameters

Color (CIELa*b*) was measured in all samples to determine changes after processing compared to the control sample. The color was measured in quintuplicate as L*, a*, and b* with a Dr. Lange portable colorimeter. The chroma and °hue values were calculated with Equations (3) and (4), respectively. °Hue values were converted from radians to degrees and plotted using SigmaPlot v12.0 (Systat Software, Inc., San Jose, CA, USA).

(3) $$\mathrm{Chroma}=\sqrt{\left(a{*}^{2}+b{*}^{2}\right)}$$

(4) $${}^{\circ}\mathrm{Hue}=\arctan\left(\frac{b*}{a*}\right)$$

### 3.7. Rheological Parameters

The apparent viscosity of processed mango nectar was estimated by application of linearly increasing shear rate values with a Brookfield DV-III ULTRA Programmable Rheometer (Brookfield Engineering Laboratories, Stoughton, MA, USA) fitted with a No. 18 spindle. A total of 10 mL of sample was added to the sample cup and maintained at 10 °C ± 0.1 with a recirculating water bath, using a mixture of distilled water and glycol. Shear rate values were variated linearly (2.64–66 s^−1^) to progressively measure the corresponding shear stress and viscosity. The apparent viscosity for each sample was reported in mPa∙s.

### 3.8. Design of Experiments and Statistical Analysis

**Figure 3:**
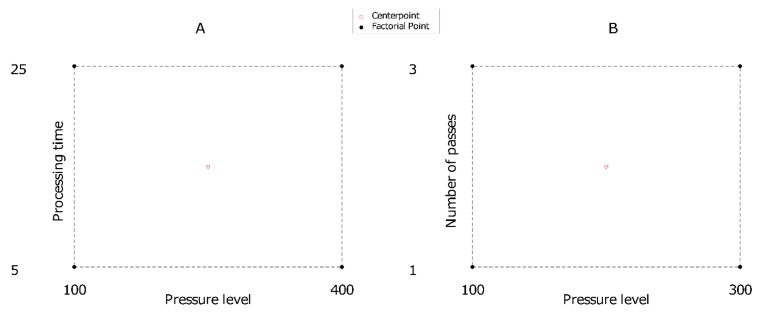
Experimental domain for hydrostatic and dynamic pressure treatments: (**A**) hydrostatic, (**B**) dynamic.

A 2^k^ experimental design was used for each type of high-pressure processing. For hydrostatic pressure, a 2^2^ design with pressure level (100 and 400 MPa for low and high levels, respectively) and processing time (5 and 25 min for low and high processing times, respectively) as experimental factors was utilized (Figure 3A). For dynamic pressure, a 2^2^ design with pressure level (100 and 300 MPa for low and high levels, respectively) and the number of passes (1 and 3 for low and high number of passes, respectively) as experimental factors was utilized (Figure 3B). The center point was independently replicated three times for each design. All data were analyzed in the Minitab 17 statistical software (Minitab, LLC, State College, PA, USA).

## 4. Conclusions

High hydrostatic and dynamic pressure processing are two interesting technologies for the preservation of fruit-based beverages like mango nectar. Regarding enzyme inactivation, our results suggest that neither is suitable for controlling the activity of pectin methylesterase without the assistance of thermal processing. Regarding polyphenol oxidase activity, high-pressure homogenization showed a good activity reduction at 300 MPa with three passes, with a possible synergy of high temperature due to adiabatic heating, even for very short holding times. For retention of quality attributes, both technologies showed good results concerning color retention. In terms of viscosity and fluid behavior, high-pressure homogenization showed interesting results at low-pressure levels, which could be useful as a tool for modifying the texture and consistency of fruit-based beverages with high pulp content. Despite these results, other relevant emergent technologies suitable for liquid food preservation such as pulsed electric fields, high-power ultrasound, high-pressure carbon dioxide, and ultraviolet irradiation could be compared in terms of effectiveness to high-pressure technologies. Additionally, the combination (hurdle effect) and optimization of emergent technologies might be a future trend for the food industry to produce the best food products with the best quality attributes and shelf-life.

## Figures and Tables

**Table 1:** Effect of hydrostatic and dynamic high-pressure treatments on the relative activity of pectin methylesterase and polyphenol oxidase in “Ataulfo” mango nectar.

**High Hydrostatic Pressure** **(MPa)**	**Processing Time** **(min)**	**Relative Enzymatic Activity (%)**	
		**Pectin Methylesterase**	**Polyphenol Oxidase**
Control	0	100.00 ± 4.84 ^a,^*	100.00 ± 7.09 ^d^
100	5	72.60 ± 1.79 ^c^	337.04 ± 48.02 ^c^
	25	96.79 ± 6.27 ^ab^	382.64 ± 37.66 ^bc^
250	15	94.92 ± 2.76 ^ab^	481.36 ± 23.99 ^a^
250	15	98.19 ± 2.80 ^a^	448.26 ± 49.81 ^ab^
250	15	99.2 ± 2.76 ^a^	398.23 ± 34.78 ^bc^
400	5	90.05 ± 3.03 ^b^	380.87 ± 33.24 ^bc^
	25	89.28 ± 2.92 ^b^	421.02 ± 18.88 ^ab^
**High Dynamic Pressure** **(MPa)**	**Number of Passes**	**Relative Enzymatic Activity (%)**	
		**Pectin Methylesterase**	**Polyphenol Oxidase**
Control	0	100.00 ± 4.84 ^ab^	100.00 ± 7.09 ^c^
100	1	104.98 ± 4.93 ^ab^	158.80 ± 7.60 ^ab^
	3	93.13 ± 3.44 ^b^	149.87 ± 11.16 ^b^
200	2	97.25 ± 4.93 ^b^	112.65 ± 7.57 ^c^
200	2	101.08 ± 7.78 ^ab^	142.34 ± 14.43 ^b^
200	2	95.13 ± 9.89 ^b^	179.43 ± 5.33 ^a^
300	1	119.95 ± 14.09 ^a^	26.74 ± 3.25 ^d^
	3	99.53 ± 4.36 ^b^	10.73 ± 1.13 ^d^

* Mean pairwise comparisons by Tukey’s test (α = 0.05). Means that do not share a letter are significantly different. Grouping information corresponds to each column.

**Table 2:** Temperature of mango nectar samples processed by high-pressure homogenization after the high-pressure valve.

Treatment	Temperature (°C) *
100 MPa/1P	31.8 ± 1.1
100 MPa/3P	37.5 ± 1.4
200 MPa/2P	58.3 ± 1.1
200 MPa/2P	55.1 ± 1.7
200 MPa/2P	52.6 ± 0.9
300 MPa/1P	65.3 ± 3.4
300 MPa/3P	73.3 ± 1.2

* Temperature values are means ± standard deviation.

**Table 3:** Effect of high hydrostatic and dynamic pressures on color parameters (CIEL*a*b*) and total color difference in ‘Ataulfo’ mango nectar.

**Hydrostatic Pressure Level** **(MPa)**	**Processing Time** **(min)**	L*	a*	b*	**ΔE**
Control	0	34.93 ± 0.37 ^a,^*	2.48 ± 0.31 ^a^	14.68 ± 0.50 ^b^	0.00 ^d^
100	5	33.15 ± 0.52 ^b^	1.75 ± 0.08 ^b^	14.14 ± 0.53 ^b^	2.18 ± 0.30 ^b^
	25	35.96 ± 0.36 ^a^	3.04 ± 0.32 ^a^	11.38 ± 0.56 ^c^	3.28 ± 0.43 ^a^
250	15	34.97 ± 1.06 ^a^	2.48 ± 0.56 ^a^	13.64 ± 1.34 ^b^	1.30 ± 0.24 ^c^
250	15	34.96 ± 0.45 ^a^	2.81 ± 0.09 ^a^	13.62 ± 0.74 ^b^	1.13 ± 0.15 ^c^
250	15	32.75 ± 0.98 ^b^	2.68 ± 0.26 ^a^	16.30 ± 1.15 ^a^	3.25 ± 0.55 ^a^
400	5	33.62 ± 0.17 ^b^	2.55 ± 0.20 ^a^	15.22 ± 0.19 ^ab^	1.54 ± 0.06 ^bc^
	25	33.36 ± 0.51 ^b^	1.87 ± 0.12 ^b^	14.56 ± 0.66 ^b^	1.67 ± 0.21 ^bc^
**Dynamic Pressure Level** **(MPa)**	**Number of Passes**	L*	a*	b*	**ΔE**
Control	0	34.93 ± 0.37 ^d^	2.48 ± 0.31 ^ab^	14.68 ± 0.50 ^d^	0.00 ^d^
100	1	38.66 ± 0.48 ^ab^	1.78 ± 0.18 ^cd^	13.96 ± 0.58 ^d^	4.10 ± 0.30 ^bc^
	3	37.02 ± 1.27 ^bcd^	2.00 ± 0.35 ^bc^	16.13 ± 1.12 ^bcd^	3.42 ± 0.40 ^c^
200	2	38.43 ± 0.73 ^abc^	2.79 ± 0.15 ^a^	15.32 ± 1.01 ^cd^	3.73 ± 0.40 ^c^
200	2	39.87 ± 0.95 ^a^	1.97 ± 0.26 ^bc^	14.57 ± 0.46 ^d^	5.34 ± 0.60 ^a^
200	2	36.95 ± 1.61 ^bcd^	1.36 ± 0.41 ^d^	17.59 ± 1.74 ^abc^	3.71 ± 0.27 ^c^
300	1	36.60 ± 1.71 ^bcd^	1.30 ± 0.38 ^d^	18.22 ± 2.21 ^ab^	3.92 ± 0.19 ^c^
	3	36.36 ± 0.26 ^cd^	1.59 ± 0.08 ^cd^	19.15 ± 0.27 ^a^	4.78 ± 0.17 ^ab^

* Mean pairwise comparison by Tukey’s test (α = 0.05). Means that do not share a letter are significantly different. Grouping information corresponds to each column.

**Table 4:** Viscosity at select shear rates of nectar samples processed by high hydrostatic pressure and high pressure homogenization.

	Nectar Viscosity (mPa∙s) at Low Shear Rate (7 s^−1^)	Nectar Viscosity (mPa∙s) at High Shear Rate (53 s^−1^)
**High Hydrostatic Pressure**		
Control	174.7 ± 8.1 ^a,^*	36.2 ± 0.2 ^ab^
100 MPa 5 min	183.0 ± 20.8 ^a^	36.6 ± 0.4 ^b^
100 MPa 25 min	201.5 ± 31.8 ^a^	40.3 ± 7.2 ^a^
250 MPa 15 min	173.9 ± 12.4 ^a^	38.7 ± 0.4 ^ab^
250 MPa 15 min	172.7 ± 11.8 ^a^	41.7 ± 1.6 ^ab^
250 MPa 15 min	177.3 ± 9.1 ^a^	37.5 ± 2.8 ^ab^
400 MPa 5 min	189.4 ± 16.7 ^a^	44.3 ± 0.6 ^a^
400 MPa 25 min	187.1 ± 29.5 ^a^	32.2 ± 0.4 ^b^
**High Pressure Homogenization**		
Control	174.7 ± 8.1 ^b^*	36.2 ± 0.2 ^b^
100 MPa 1P	281.2 ± 14.3 ^a^	39.4 ± 0.2 ^a^
100 MPa 3P	221.0 ± 5.9 ^ab^	26.5 ± 0.6 ^c^
200 MPa 2P	172.4 ± 7.3 ^b^	25.6 ± 0.7 ^c^
200 MPa 2P	165.2 ± 6.2 ^b^	24.8 ± 0.4 ^c^
200 MPa 2P	179.7 ± 5.8 ^b^	26.3 ± 0.5 ^c^
300 MPa 1P	166.6 ± 7.7 ^b^	26.6 ± 0.8 ^c^
300 MPa 3P	185.8 ± 8.6 ^b^	24.9 ± 0.1 ^c^

* Mean pairwise comparison by Tukey’s test (α = 0.05). Means that do not share a letter are significantly different. Grouping information corresponds to each column.

## Data Availability

Not applicable.
